# Changing trend of caries from 1989 to 2004 among 12-year old Sardinian children

**DOI:** 10.1186/1471-2458-7-28

**Published:** 2007-03-01

**Authors:** Guglielmo Campus, Gianluca Sacco, MariaGrazia Cagetti, Silvio Abati

**Affiliations:** 1 Dental Institute, University of Sassari, Viale San Pietro 43/c I-07100 Sassari, Italy; 2 School of Dentistry, University of Milan, "S.Paolo Hospital" WHO Collaborating Centre of Milan for Epidemiology and Community Dentistry, Via Beldiletto 1 Milano, Italy

## Abstract

**Background:**

During the past decades, the prevalence of caries disease in the population of Western industrialized countries has decreased markedly. In children also, a reduction of dental caries experience has been reported by many authors. The aim of this paper was to evaluate the trend of dental caries prevalence in 12-year-old children living in the city of Sassari, (Italy), by five cross-sectional studies conducted in 1989, 1992, 1995, 1998 and 2004.

**Methods:**

In all cohorts, dental caries (DMFT and SiC Index according to WHO indications), was measured. For each variable measured (DMFT and sub-indices, SiC Index), differences in proportions among the five cohorts during the fifteen years were tested using χ^2^-square test.

**Results:**

The mean DMFT index decreased from 4.3 ± 3.1 in 1989 to 0.8 ± 1.5 in 2004. The prevalence of untreated caries (DT) had a notable decrease between 1992 and 1995, increased slightly between 1995 and 1998 and had the greatest decrease in 2004. The number of filled teeth remains low. The percentage of caries-free children increased from 10% to 64%, whereas the percentage of untreated caries changed from 44% in 1989 to 62% in 2004. SiC Index decreased from 7.8 in 1989 to 3.9 in 2004.

**Conclusion:**

On the basis of the results of DMFT and SiC Index, caries experience has been reduced. The vigilance and the promotion of a higher standard of personal oral hygiene and dental check-ups are necessary to obtain an improvement of oral status in the future adult population and to reach the new WHO global goals.

## Background

Dental caries remains the single most common disease of childhood that is not self-limiting or tractable with antibiotics. During a relatively short period of time (about twenty-five years), dental caries has undergone to a striking reduction in most industrialized countries [1,2]. Many factors played a role in this decrease. Most authors agree that the widespread use of fluoride in the toothpaste is the main reason for this decrease [3,4]. Preventive programs and changes in the restorative dental treatment approach are reported as important factors, too [5-7]. However, the real contribution of health services in the improvement of oral health, remains unclear. A possible contribution of the dental services to the caries decrease is the change in the diagnostic and treatment criteria [8]. The improvement in oral hygiene and the natural cyclical variation may also explain the decrease. The mass media and the advertisement bring new standard for the every-day life and they have a strong influence on both dietary and dental care habits. Additional factors like overall nutrition, number of meals and snacks per day, use of noncariogenic sweeteners and socioeconomic status are to be considered [8].

A large analysis, assessing the role of dental care in the decrease of caries in 12-year-old children in 18 industrialized countries, has shown that dental service were not important in explaining the disease level decrease [1,8,10,11]. Countries with very different population's ethnicity, dentist/patient ratios and preventive dental care systems, had very similar 12-year-old caries experience. In Italy, comprehensive public oral health care is provided, free of charge, only for children under the age of 6. However, neither preventive treatments or preventive care programs are supplied at the national level. Despite this, a remarkable improvement in dental health of children and adolescents has been observed in recent years [12-17].

The aim of the present survey was to compare the changes in the prevalence of caries, during the period from 1989–2004, in 12-year old children living in Sassari, Sardinia, Italy.

## Methods

### Study population

Sassari is the second city of Sardinia with 124,929 inhabitants [18]. Several epidemiological surveys were performed. Cohorts consisted of twelve-year-old children living in the inner-city area of Sassari. The fluoride concentration in the tap water is low, varying from 0.05 to 0.40 ppm [19].

Sampling was conducted at the school district level. The number of children examined in each of the five cohorts is presented in Table 1.

**Table 1:** Number of children examined in 1989, 1992, 1995, and 1999, 2004.

Year	No children	No boys	No girls
1989	372	182	190
1992	316	154	162
1995	410	209	201
1999	403	206	197
2004	301	134	167

In 1989, all 15 school districts were invited to participate in the survey. Five school districts, with a total of 398 children agreed to participate. 372 out of 398 children took part in the survey. In 1992, all 15 school districts were contacted again. Five of them agreed to participate in the survey. 316 children were recruited out of 372. In 1995, our survey was part of a Europe-wide study [20]. Three out of the 12 school districts agreed to participate in the study and a total of 410 children were selected. In 1999 and 2004, the children were recruited using systematic cluster sampling: every class was identified as a cluster and compiled into a list. The first cluster in the list was randomly chosen while the others were selected systematically at intervals of four classes. The number of subjects in each class was approximately the same. 403 and 301 students, participated in the 1999 and 2004 surveys, respectively.

Parents of the children were informed about the study and invited to participate by letter or direct interview at the school.

### Ethics

The ethical principles defined by World Medical Association Declaration of Helsinki were followed in this study and all parents of the children gave written informed consent. Approval for the study was granted by the Ethics Committee of the Faculty of Medicine, The University of Sassari, Italy.

### Data collection

All the examinations were carried out by two examiners in the Dental Institute of University of Sassari, using a plane mirror and a CPI-ball probe under optimal light, after air-drying. Decayed, Missing and Filled Teeth (DMFT index) were recorded following the WHO criteria [21]. A tooth was classified healthy if it showed normal enamel translucency after drying; while a cavitated active lesion was classified as a cavity in enamel or enamel and dentine with soft consistency and light-brown coloration.

To focus attention to the subjects most affected by caries, the SiC Index "Significant Caries Index" was also calculated. The SiC Index is a fairly precise statistic for assessing caries in the most caries-prone third of sample. Another advantage of the SiC Index is: in population with low caries experience, minute white fillings are frequently placed in pits and fissures. Part of these small restorations could likely to be overlooked. On the contrary, the subjects that constitute the upper tertile providing the SiC Index, have many extensive restorations and cavities, which are rarely overlooked. This means that in situations of low caries prevalence, the overall DMFT has a higher risk of underestimation than the SiC Index [11,22,23].

### Statistical analysis

All analyses were performed using Stata 8.2 (Statacorp. USA). For variables regarding clinical caries disease (DMFT index and subgroups DT, MT and FT) the mean and standard deviation of number of affected teeth was calculated. Differences in mean score among the cohorts were tested using the analysis of variance (ANOVA). At the beginning of the study (1989) the inter-examiner reliability was assessed for DMFT using the kappa statistics. In 1995–2004 this test was performed only for the DT component. The inter- and intra-examiner consistency of diagnostic judgements (*n = 25 for each cohort*), calculated as Cohen's kappa and regarding the DMFT index and/or components, suggested a good agreement (range values 0.78–0.86).

All analyses were performed using a significance level of 0.05.

## Results

One thousand eight hundred and two children (1802), 885 (49.1%) boys and 917 (50.9%) girls were enrolled in the different surveys (Table 1). The mean values of DMFT and components are presented in Table 2. The mean DMFT index decreased from 4.3 ± 3.1 in 1989 to 0.8 ± 1.5 in 2004 (ANOVA one way p < 0.001). No statistically differences were seen between gender in both, caries level and prevalence of the disease.

**Table 2:** Mean value ± standard deviation, (skewness index) of DMFT index and components in 1989, 1992, 1995, and 1999, 2004.

Year	DMFT	DT	MT	FT
1989	4.3 ± 3.1 (γ1 = 0.49)	1.9 ± 2.1	0.1 ± 0.5	2.2 ± 2.4
1992	4.1 ± 3.4 (γ1 = 0.90)	2.1 ± 2.5	0.1 ± 0.6	1.9 ± 2.6
1995	3.0 ± 2.8 (γ1 = 1.20)	1.5 ± 2.3	0.1 ± 0.1	1.4 ± 1.8
1999	2.3 ± 2.7 (γ1 = 1.31)	1.7 ± 2.2	0.05 ± 0.1	0.7 ± 1.4
2004	0.8 ± 1.57 (γ1 = 1.45)	0.5 ± 1.04	0.02 ± 0.2	0.3 ± 0.9
*Anova one way*	*p < 0.01*	*p < 0.01*	*p < 0.01*	*p < 0.01*

**Figure 1:**
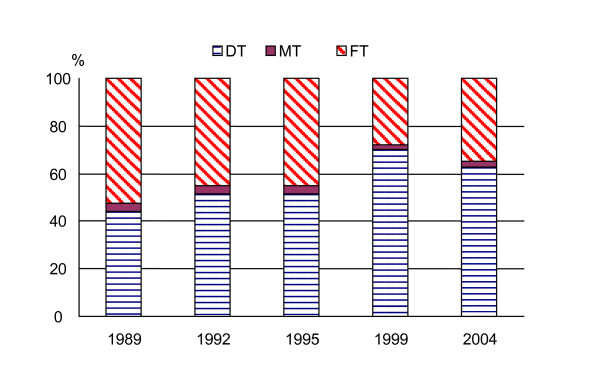
Variation of DT, MT and FT components in DMFT index.

The FT component decreased from 2.2 to 0.3. In 1989, about 52% of the DMFT was due to the FT, while in 1998 only about 35% was attributable to this component (Fig. 1). Instead the D component increased from 44% to 62%.

The percentage of caries-free children increased from 10.5% in the 1989 survey to 63.8% in 2004 (p = 0.01). Conversely, the percentage of DT- free children increased from 35% in 1989 to 72 % in 2004, whereas the percentage of children with a DMFT >10 decreased from 3% to almost 1% (Table 3).

**Table 3:** Distribution of children, as percentage, by year, according to the DMFT and DT.

	DMFT				DT			
	0	1–5	6–10	>10	0	1–5	6-1	>10

1989	10.5	57.0	29.6	2.9	35.5	57.5	7.0	0.0
1992	15.6	54.3	27.3	2.8	34.3	55.0	10.7	0.0
1995	27.6	55.3	15.7	1.4	48.6	44.7	5.7	1.0
1999	38.2	48.9	11.9	1.0	46.9	45.2	7.4	0.5
2004	63.8	29.2	6.0	1.0	71.8	27.9	0.3	--

χ^2 ^= 12.3 p = 0.01 χ^2 ^= 9.4 p < 0.05

SiC Index is shown in table 4; SiC value decreased from 7.8 in 1989 to 3.9 in 2004. The percentage of children with a SiC Index >3 lowered too (from 32.8% in 1989 to 12.4% in 2004, p < 0.001).

**Table 4:** SiC Index, descriptive statistics: number of subject, mean value ± standard deviation. Number (percentage) of subjects with SiC Index >3.

Year	number of subjects	SiC mean ± SD	SIC>3 n (%)
1989	123	7.8 ± 0.7	123 (33.0)
1992	104	7.4 ± 1.2	96 (30.5)
1995	135	5.6 ± 1.8	77 (18.7)
1999	133	5.5 ± 2.1	71 (17.5)
2004	99	3.90 ± 2.8	37 (12.4)
		*Anova one way *p < 0.001	χ^2 ^p < 0.01

The percentage of subjects with a SIC>3 was calculated on the total of the cohort.

## Discussion

Epidemiology is fundamental in dental care planning. In this series of epidemiological studies it was possible to estimate in detail the changes in caries prevalence over a 15-year period in a defined children Italian population.

Burt [24] describes the benefits of cross-sectional data. When these surveys are repeated periodically under generally similar conditions (like these surveys), broad oral health trend over time can be estimated. Sassari is too small to allow community dentists to generalize results to the whole country. Nevertheless, the results observed showed that the onset and development of dental disease decreased during the fifteen-year period. Caries prevalence still remains at a high level for a certain percentage of subjects. The percentage of caries-free children increased significantly. It is important to note the fluoride concentration in the drinking water is low [19].

Mean DMFT value decreased dramatically from over four in 1989 to less than one in 2004, achieving an important result for oral health in children. The World Health Assembly set the global average for dental caries experience in 12-year-old children should not be higher than 3.0 in the year 2000 and a DMFT ≤ 1 for 2010 [25]. The cohorts surveyed, already achieved this goal.

In this kind of research, the level of treatment needed can only be assessed in very broad terms. This is may be due to the difference in the evaluation criteria of patient care used by the epidemiologists and the ones used by the dentists [7,26-29]. One of the limitations of the DMF index is that it is based on the assumption that all filled teeth were carious prior to the filling. This assumption may lead to overestimating caries experience as expressed by the FT component of the DMFT [29]. In the five surveys the FT index decreased about of 90%; so the contribution of the component to the prevalence of caries changed drastically. In 1989, the FT component was 52% of the DMFT index, while in 2004 the FT component was only 35%. In the same time, the disease activity component (D index) increased from 44% to 62 per cent. This dramatic decrease of dental treatment could be linked to the economic crisis during the years 1994–1997.

Another limitation of DMFT index is the absence of a denominator. This implies that DMFT values should be age-adjusted to be meaningfully interpreted [10,22,29,30]. No age-adjustment was performed, because, all subjects were in the same age-range at the moment of the examination. Nonetheless, DMFT index remains the basis for caries measurement.

The distribution of the DMFT was reasonably symmetrical in 1989 with a mode of 4. About 10% of sample had a DMFT of zero. In 2004, the distribution was almost unimodal with the mode near zero. It is known that the asymmetrical distribution of the index DMFT has an important epidemiological significance: while most of the children are caries-free, a small group still present high score of caries disease. Almost two third of the sample had a DMFT index = 0. Following Spencer's theory [29], some of this skewness may be the result of misleading in diagnostic criteria for classifying cavity lesions. Traditionally, cavity lesions were identified on the postulate that such lesion required a restoration. The recent change in treatment option due to new restoration materials, calls for new and more complex system for observing and recording the carious process. Although this could be not a problem in these cohorts, it was observed a decrease in the DT component between 1989 and 1995, with a slight increase in 1998 and a new reduction in 2004.

Parallel to the decrease in caries prevalence, the majority of sample has DMFT value 0, 1 or 2, whereas a minority of them still has high level of caries. The decrease of the SiC Index demonstrates that, even in the sample with the highest caries experience, nowadays often labeled as high risk group, caries experience has been reduced substantially. A similar reduction was observed in Switzerland [11] with a decrease of more than 80% (1964–1996). Since 1996 SiC has remained below the upper limit of 3.0, gaining so the WHO goal for 2015. In the present study sample, in 2004, this goal appears reachable.

A study limit is indeed in the different sampling technique used in the five surveys. A more systematic sampling would have yielded a higher percent of participants. Moreover, the school districts that did not agree to participate in the surveys, were similar, demographically and social-economically, to the districts that agreed to participate. Thus, children who took part at the surveys could be representative of all the children in the same age group. All children enrolled in each examined cohort, lived in the inner-part of the town, deleting so the bias effect of urbanization on caries disease [30]; therefore, no selection or sampling bias was introduced.

## Conclusion

This study documents for the first time a caries trend in Italy and shows an improvement in dental health among the majority of Sardinian children in 2004 compared to the earlier years. However, at the same time public health dentistry have to further develop and pursue a high-risk approach and still maintains vigilance to prevent a delayed caries development in the future adult population.

## Competing interests

The author(s) declare that they have no competing interests.

## Authors' contributions

GC participated in the design of the study, carried out the acquisition of data; performed the statistical analysis and was involved in drafting the manuscript;

GS carried out the acquisition of data; and was involved in drafting the manuscript;

MGC conceived of the study and participated in its design.

SA conceived of the study and participated in its design.

All authors read and approved the final manuscript.

## Pre-publication history

The pre-publication history for this paper can be accessed here:


